# Idiopathic Granulomatous Hypophysitis Mimicking Pituitary Abscess

**DOI:** 10.1097/MD.0000000000001099

**Published:** 2015-07-17

**Authors:** Xiangyi Kong, Renzhi Wang, Yi Yang, Huanwen Wu, Changbao Su, Wenbin Ma, Yongning Li, Bing Xing, Wei Lian, Zhiqin Xu, Yong Yao, Zuyuan Ren

**Affiliations:** From the Department of Neurosurgery (XK, RW, YY, CS, WM, YL, BX, WL, ZX, YY, ZR), Peking Union Medical College Hospital, Chinese Academy of Medical Sciences; and Department of Pathology (HW), Peking Union Medical College Hospital, Chinese Academy of Medical Sciences, No. 1 Shuaifuyuan Hutong of Dongcheng District, Beijing, P. R. China.

## Abstract

Idiopathic granulomatous hypophysitis (IGH) is a rare inflammatory disease of the pituitary that commonly presents with enlargement of the pituitary gland. Clinically and radiologically, IGH is a rare sellar entity easily to be misdiagnosed as a pituitary adenoma.

Through such a case, we aim to present this rarity and emphasize the importance to correctly diagnose confusing pituitary lesions comprehensively by clinical presentations, radiological signs, and biopsy.

We present an uncommon case of IGH in a 19-year-old man. The patient was admitted to the hospital with severe headache, vomiting, and vision's sharp decline. Magnetic resonance imaging showed a sellar lesion with obvious cystic change and ring enhancement. The disease course including diagnosis and treatment was presented and analyzed in detail. The pertinent literature is reviewed regarding this uncommon entity.

The patient underwent surgical exploration and partial resection via the transsphenoidal approach. The pathologic findings suggested IGH giving no significant evidences of systemic granulomatous disease and venereal disease. Large dose methylprednisolone was then used. The pituitary function recovered, but there was no apparent improvement of his vision.

IGH is a rarely occurred inflammatory disease of unknown etiology. It is difficult to diagnose preoperatively and is often misdiagnosed. Although rare, IGH should be kept in mind in terms of differential diagnosis of sellar region lesions.

## INTRODUCTION

Hypophysitis is relatively a rare disorder, with an estimated incidence of 1 case per 9 million people per year and imaging diagnosis is difficult.^1^ They encompass a wide spectrum of pathology including lymphocytic hypophysitis (LH), granulomatous hypophysitis (GH), local manifestations of systemic disease, and a multitude of infectious processes.^2^ GH is a chronic inflammatory condition of the pituitary, first described by Simmonds in 1917.^3^ Majority of pituitary granulomas represent a specific lesion such as syphilis, tuberculosis, sarcoidosis, or histiocytosis-X. In absence of any demonstrable causative agent, the process is termed idiopathic granulomatous hypophysitis (IGH). The most common presenting symptom was headache, followed by chronically progressive visual changes. Magnetic resonance imaging (MRI)-specific data for IGH was poorly reported, with pituitary enlargement the most common feature. Contrast enhancement and pituitary stalk thickening may also appear. Clinically and radiologically, IGH is a rare sellar entity easily to be misdiagnosed as a pituitary adenoma.

Herein, we present a case of a 19-year-old man suffering from IGH complaining of vision's sharp decline to blindness within 1 week. Uniquely, the MRI showed a sellar lesion with obvious cystic change and ring enhancement, very much mimicking a pituitary or peripituitary abscess. To the best of our knowledge, this is the first reported case of IGH presenting in this manner giving his unique clinical and image features. Because the rarity of IGH often leads to the misdiagnosis of either another inflammatory and infectious disease or a pituitary tumor, we also review the latest literature on the etiology, diagnosis, and treatment of IGH.

## CASE REPORT

**FIGURE 1:**
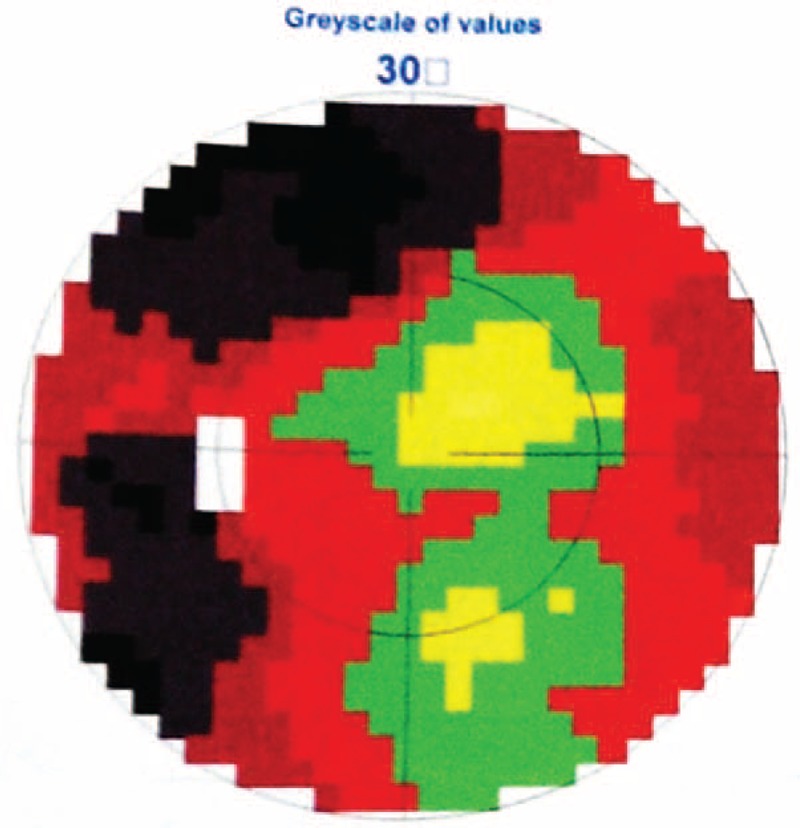
Visual field test of the left eye showed temporal incomplete hemianopia (black areas) and nasal scotomas (crimson areas).

**FIGURE 2:**
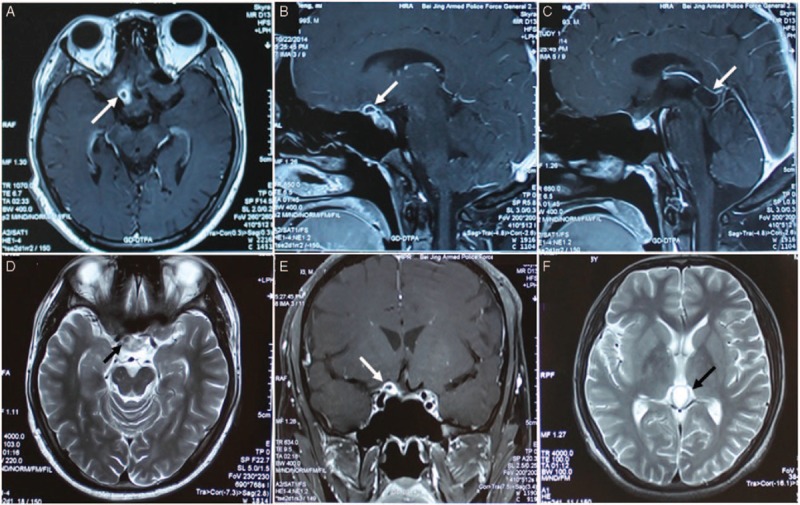
Radiological evaluation of the brain before surgery. T1-weighted axial gadolinium-enhanced MRI demonstrated a ring-enhancing cystic lesion at the right part of suprasellar region in the anterior of optic chiasma (arrows) (A, C, D). T2-weighted image demonstrates the same lesion as in the previous image (arrow) (B). A cyst in the pineal region was also showed with long-T1 and long-T2 signals (arrows) (E, F).

A 19-year-old Chinese man, without particular previous medical history, had complained of a bilateral temporal headache for more than half a year. The pain was diffuse, dull, and paroxysmal, and could be alleviated by rest. He also presented with transient fever (maximum 39.2°C) for several times. He did not seek medical services until nausea and vomiting occasionally occurred especially in the morning 1 month after the onset. He underwent a head computed tomography scan at the local hospital, showing a 15 mm × 10 mm cyst in the pineal region (just a chance finding). No apparent changes were found in other intracranial structures including the sella turcica area. He was treated pharmacologically with analgesics. However, his condition did not improve. After 2 weeks, because of frequent nausea and vomiting, he came to our hospital. The patient was down in spirits with heavy complexion. In addition to the above symptoms, he also complained a mildly decreased bilateral visual acuity (VA) in recent weeks. He denied polyuria, polydipsia, and sexual hypoactivity, and had no symptoms of unconsciousness, convulsion, epilepsy, and cognitive disorder, and no special circumstances regarding his family history or personal history related to his presentation were identified. Neurological examination showed a normal function of both sensation and motor at 4 limbs. No apparent changes in superficial and deep tendon reflexes were observed. Both superficial and deep tendon reflexes were normal. Pathological signs and ataxia were absent. Upon rough ophthalmologic examination, his left palpebral fissure was relatively small with ptosis, and pupillary light reflex was insensitive on the right side. The left VA was 0.8 and the right 0.6. There was suspicious defect in right upper quadrant of his right eye. However, his VA declined sharply. Just within 3 days while waiting for his head MRI scanning, the patient complained he even could not see anything with his right eye, and his vision on the left side blurred further. Upon specialized ophthalmologic examination, relative afferent pupillary defect on the right side was strong positive and fundoscopic examination was unremarkable. Temporal incomplete hemianopia and nasal scotomas of his left eye were observed (Figure 1). His right VA was too low to undergo a visual field (VF) test. Head MRI revealed a nodular lesion (12 mm × 10 mm × 8 mm) at the right part of suprasellar region in the anterior of optic chiasma. The lesion's interior was cystic, which was isointensity as gray matter on T1-weighted and T2-weighted images. After injection of an intravenous contrast agent, the lesion was obviously ring-enhanced. The lesion circumvented the right internal carotid artery (C7 segment) and had no clear limit with visual chiama. In addition, the pituitary stalk, funnel, right optic nerve, the back of the right gyrus rectus, and substantia perforata anterior were also involved (Figure 2). These images were representative of the features of a pituitary or peripituitary abscess. Lumbar puncture showed a normal intracranial pressure at 130 mmH_2_O. The cerebrospinal fluid (CSF) was clear. Total cell count in CSF was 17 × 106/L, and white blood cell was 16 × 106/L, with mononuclear lymphocytes and phagocytes the majority. CSF biochemical analysis yielded no abnormal finding. Endocrinological evaluation showed a normal thyroid and adrenocortical function and a mild hyperprolactinemia (prolactin [PRL] 16.39 ng/mL, normal: 2.64–13.13). Immunological and rheumatic indicators were all within the normal range.

**FIGURE 3:**
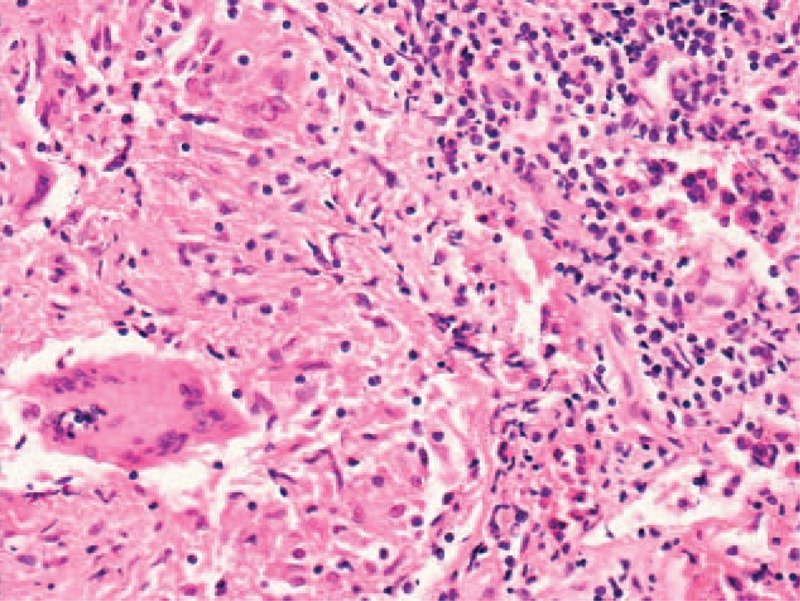
Histopathologic test (hematoxylin-eosin staining, 200×) revealed granulomatous changes with multinucleated giant cells and macrophages infiltration (on the bottom left of the field area). On the right was the surrounding adenohypophysial tissue.

The patient's vision continued to deteriorate. On the 6th day after his admission, he could only see his hand move with his right eye, and his left eye could only see light from the outside margin of the eyelid. He underwent surgical exploration via the transsphenoidal approach. Only partial resection was achieved. The pathologic findings revealed granulomatous changes with multinucleated giant cells and macrophages infiltration (Figure 3). Once the histopathologic diagnosis was clear and definite, the patient was further undergone cutaneous, skeletal, visceral, and laboratory examinations for systemic granulomatous disease such as tuberculosis, syphilis, sarcoidosis, brucellosis, and histiocytosis-X. Owing to no significant evidences of systemic granulomatous disease and venereal disease were found, the patient was finally diagnosed with IGH. Giving the resection was limited, impulsion therapy with large dose methylprednisolone was then used after the final diagnosis. The patient's headache and vomiting have been completely cured, and his Pituitary function is primarily normal after surgery, but there is no apparent improvement of his vision. He is now under outpatient follow-up care.

## DISCUSSION

Inflammatory diseases involving the pituitary gland are rare when compared with adenomas. Histologically, inflammatory hypophysitis is classified into 5 subtypes: lymphocytic, granulamatous, xanthomatous, xanthogranulomatous, and necrotizing.^4,5^ Hypophysitis can also be classified as idiopathic hypophysitis and secondary hypophysitis which may be caused by systemic inflammatory disorders such as tuberculosis, Wegener's granulomatosis, histiocytosis-X, and sarcoidosis.^4,6^ The majority of inflammatory pituitary lesions occur in women, are related with the postpartum period, and most of them are in the form of LH.^7^

IGH, an extremely rare chronic pituitary inflammation, has been reported in <1% of sellar lesions based on surgical findings with the transsphenoidal approach.^8^ Although no definite etiology has been described, there are differing perspectives on the pathogenesis of IGH. The mainstream view that the pathogenesis of IGH was thought to be attributed to autoimmunity.^8^ However, in our case, no autoimmune background was found. The relationship, if any, between IGH and LH is unclear.^9,10^ Some authors feel these conditions are different or are opposite ends of the spectrum of same disease, with fibrosis representing the end stage of the inflammatory process.^5^ Others suggest IGH differs from LH in certain epidemiological features, which are mostly associated with pregnancy and autoimmune diseases. A recent systematic review of 82 cases of IGH demonstrated a female predilection of IGH, and showed that most cases of IGH exhibit lymphocytic infiltration of the pituitary gland. In addition, the mean age of presentation in IGH is 43 years, some 8 years subsequent to the mean age of presentation in LH, suggesting the former view mentioned above.^11^

Headaches are the most common presenting symptom of GH.^11^ Patients may also present with chronically progressive chiasmal compression, hypopituitarism, amenorrhea-galactorrhea, hyperprolactinemia, fatigue, and diabetes insipidus.^10^ In our case, the patient demonstrated a sudden onset of severe blurred vision and VF's sharply increased defect, which is different from previous reports. On MRI, there was no clear limit between the IGH lesion and the thickening visual chiama and right optic nerve. Vision's sharp decline suggested the lesion developed rapidly and furiously invaded the visual pathway. In addition, lateral expansion of pituitary mass into the cavernous sinus could compress III, IV, or VI cranial nerves, resulting in diplopia and subsequently ocular misalignment. The patient's ptosis was mostly secondary to oculomotor paralysis because of pressure and stimulation. Given this, we once considered a possible diagnosis of a rapidly developing optic glioma, for optic glioma typically shows partial or total loss of vision or changes in the optic nerves and nausea and vomiting may also be present.^12^ On MRI, it shows as an enlargement, kinking, and buckling of the optic nerve, and may also appear cystic degeneration.^13^ However, optic nerve glioma is a rare kind of cancer, usually slow-growing and found in children. It is rarely found in individuals over the age of 20. Apart from the visual impairment, the patient also demonstrated a mild hyperprolactinaemia, which may due to the compression of the pituitary stalk or the inflmmatory process itself preventing the inhibitory regulation of PRL release by hypothalamic dopamine.^14^

Imaging appearances of different types of primary hypophysitis are very similar, and it is really difficult to differentiate hypophysitis from the commonest sellar region lesion pituitary adenoma just based on the MRI which is far from specific enough.^5^ But in most clinical situations, as for hypophysitis, the pituitary enhancement is usually intense, homogeneous, and symmetrical, and the invasive changes of the sella turcica floor are rare.^15^ It is even harder to diagnose IGH preoperatively; available diagnostic clues on MRI are that the neurohypophysis’ bright spot is rarely seen and pituitary stalk usually gets thicker.^11^ Perez-Nunez^16^ once described a case of LH imageologically mimicking a pituitary abscess presenting with the MRI of a sellar cystic lesion with a hypointense core surrounded by a contrastenhancing rim, contacting with, but not compressing the optic chiasma. He concluded that LH should be kept in mind when differentiate cystic ring-enhanced cystic sellar region lesions. Our case is the second reported one of primary hypophysitis and the first reported one of IGH with cystic and ring-enhanced MRI appearance. It has 2 prominent features: it was located in the suprasellar region instead of saddle area and the pituitary itself was not enlarged and the cystic and ring-enhanced lesion adhered tightly to the optic chiasma and nerve and resulted in a precipitously worsened vision loss. In addition, a pineal cyst was hit upon.

Transsphenoidal surgery is a primary diagnostic option, especially for those whose clinical and radiological presentation is not typical and diagnosis is not confirmed.^15^ Histopathological findings of pituitary biopsy remain the gold standard for diagnosing primary hypophysitis.^10^ In fact, majority of reported IGH and approximately half of the primary hypophysitis were misdiagnosed as pituitary adenomas before surgery.^17,18^ If primary hypophysitis is suspected, an intraoperative histology on frozen sections is recommended to confirm the diagnosis and avoid extensive surgery because hypopituitarism may occur or be worsened after extensive resection.^5,18^ As the natural history of IGH is incompletely understood yet, its treatment is still controversial.^19^ Surgery not only provides live tissue for histological diagnosis, but can rapidly decompress the mass lesion, thereby resolving headache and visual deficits immediately. Hypopituitarism resolves after surgery in some cases, but many patients remain on full hormone replacement therapy. Satisfactory response to high-dose steroid therapy or anti-inflammatory and immunosuppressive (methotrexate, cyclosporine A, azathioprine) treatments have also been widely reported.^18,20,21^

What is noteworthy is that in the present case, there was no apparent improvement of the patient's vision after recovery of normal pituitary function. We guess there are at least 3 possible reasons accounting for that. First, the resection surgery was not total but partial. Although impulsion therapy with large dose methylprednisolone was used after surgery, there might still be some left lesion tissues which could still exert mass compression effect and stimulatory effect on the optic chiasm. Second, the patient's preoperative VF and VA were too poor and deteriorated rapidly. According to a multivariate analysis for pituitary adenomas, the only independent predictor of postoperative recovery of VF was the degree of preoperative VF deficit. Postoperative VF recovery was also related to both the duration of symptoms and the severity of preoperative VA. Third, the pituitary lesion nature in this case is IGH rather than the commonest adenoma, whose pathogenic effect may be not limited to simple compression. So in this circumstance, the optic chiasm could encounter some nonreversible pathologic change such as optic atrophy.^22^ All in all, VF recovery may be related to many factors including differences in the patient population studied, differences in preoperative deficits in VF and VA, the lesions’ pathological nature, the extent of lesion resection, the dose of postoperative radiotherapy, and so on.

## CONCLUSION

We report an uncommon case of suprasellar IGH with cystic and ring-enhanced MRI appearance resulting in a precipitously worsened vision loss. IGH is a rarely occurred inflammatory disease of unknown etiology. It is difficult to diagnose preoperatively and is often misdiagnosed. With this article, we not only aim to present this rare entity, but also to emphasize the importance to correctly diagnose cystic ring-enhancing sellar lesions to ensure the patient receives proper treatment.
